# Supplementary material for: Two new Cortinarius species in subgenus Leprocybe from Southwest China
Source: PeerJ. 2024 Jul 12;12:e17599. doi: 10.7717/peerj.17599 (PMC11248999; doi:10.7717/peerj.17599)
Supplement: Supplemental Information 2 [file peerj-12-17599-s002.docx]

**The note of a adding author**

Dear editor,

In this revised manuscript, we add Ming-Jun Zhao as the new author. According to the reviewers’ suggestion, we need to take some additional experiment especially replace with a new high-quality image. All the author are not poor at make the illustration, so we invite Ming-Jun Zhao to finish Figure 3. Besides, Zhao has taken the supplementary morphologic observation to solve the questions raised by reviewers. At last, Zhao help to improve the English of the manuscript. Zhao did these contributions during Feb 20 to April 15, 2024.

The list of new author contributions is listed below:

| New author | Contributions |
| --- | --- |
| Ming-Jun Zhao | 1. Participate in the additional experiment  2. Made the new illustration of Figure 3  3. Help improve the English |

Best regards,

Peng Hong, on behalf of all authors
